# A Novel Early Warning System Based on a Sediment Microbial Fuel Cell for In Situ and Real Time Hexavalent Chromium Detection in Industrial Wastewater

**DOI:** 10.3390/s18020642

**Published:** 2018-02-22

**Authors:** Shuai Zhao, Pu Liu, Yongyan Niu, Zhengjun Chen, Aman Khan, Pengyun Zhang, Xiangkai Li

**Affiliations:** 1 MOE Key Laboratory of Cell Activities and Stress Adaptations, School of Life Sciences, Lanzhou University, Tianshuinanlu #222, Lanzhou 730000, Gansu, China; zhaosh15@lzu.edu.cn (S.Z.); niuyy2014@lzu.edu.cn (Y.N.); chenzh12@lzu.edu.cn (Z.C.); aman15@lzu.edu.cn (A.K.); 2 Gansu Key Laboratory of Biomonitoring and Bioremediation for Environmental Pollution, School of Life Sciences, Lanzhou University, Tianshuinanlu #222, Lanzhou 730000, Gansu, China; liupu@lzu.edu.cn; 3 Department of Developmental Biology, School of Life Sciences, Lanzhou University, Tianshuinanlu #222, Lanzhou 730000, Gansu, China; 4 Gansu Academy of Membrane Science and Technology, Duanjiatanlu #1272, Lanzhou 730020, Gansu, China; zhangpymky@163.com

**Keywords:** sediment microbial fuel cell, hexavalent chromium, biosensor, in situ, real time

## Abstract

Hexavalent chromium (Cr(VI)) is a well-known toxic heavy metal in industrial wastewater, but in situ and real time monitoring cannot be achieved by current methods used during industrial wastewater treatment processes. In this study, a Sediment Microbial Fuel Cell (SMFC) was used as a biosensor for in situ real-time monitoring of Cr(VI), which was the organic substrate is oxidized in the anode and Cr(VI) is reduced at the cathode simultaneously. The pH 6.4 and temperature 25 °C were optimal conditions for the operation. Under the optimal conditions, linearity (R^2^ = 0.9935) of the generated voltage was observed in the Cr(VI) concentration range from 0.2 to 0.7 mg/L. The system showed high specificity for Cr(VI), as other co-existing ions such as Cu^2+^, Zn^2+^, and Pb^2+^ did not interfere with Cr(VI) detection. In addition, when the sediment MFC-based biosensor was applied for measuring Cr(VI) in actual wastewater samples, a low deviation (<8%) was obtained, which indicated its potential as a reliable biosensor device. MiSeq sequencing results showed that electrochemically active bacteria (*Geobacter* and *Pseudomonas*) were enriched at least two-fold on the biofilm of the anode in the biosensor as compared to the SMFC without Cr(VI). Cyclic voltammetry curves indicated that a pair of oxidation/reduction peaks appeared at −111 mV and 581 mV, respectively. These results demonstrated that the proposed sediment microbial fuel cell-based biosensor can be applied as an early warning device for real time in situ detection of Cr(VI) in industrial wastewaters.

## 1. Introduction

Industrial wastewater streams generally contain heavy metals such as Cr, Cu, Pb [1], among which chromium is generally the major contaminant found in industrial wastewater, which may cause health problems and pollute natural ecosystems when at high concentrations [2]. In aquatic ecosystems, trivalent [Cr(III)] and hexavalent [Cr(VI)] are its two major oxidation forms [3]. Of these Cr(VI) can cause more serious injuries than Cr(III) to plants and humans, such as skin cancer, allergies and the lung cancer [4] due to its high solubility, mobility, and mutagenicity. Several traditional treatment technologies can remove Cr(VI) efficiently, including physical, chemistry and biological methods [5], but samples treated with all of these methods need to be verified after processing to assess whether they meet the discharge standard concentration. If the Cr(VI) emission exceeds the permitted concentration, it can cause serious environmental problems and human health disorders [6]. Thus, it is crucial for the Cr(VI) concentration of effluents from industrial wastewater treatment plants to be strictly controlled in real time prior to its discharge to the environment. At present, several chemical analysis methods including spectrophotometry, atomic absorption spectrometry (AAS), and infrared-absorption spectrometry are applied to detect the Cr(VI) concentration in water samples [7]. These methods display high selectivity and sensitivity, however, the existing methods have drawbacks such as a high cost or the requirement for complicated extraction, purification, and concentration procedures [8], and they cannot provide in situ and real time values. 

Recently, biosensors, which are integrated devices that combine biological materials with a transducer, have been developed for the quantitative or semi-quantitative monitoring of various analytes [9], such as heavy metals like Cu^2+^, Hg^2+^ and Pb^2+^ [10], biochemical oxygen demand (BOD) [11] and volatile fatty acids [12]. Previous studies have reported biosensors for Cr(VI) detection based on an amperometric enzyme biosensor utilizing cytochrome c3 or urease, relying on cell biosensors using engineered strains [13,14]. Compared with traditional techniques, these biosensors exhibited simple, inexpensive and portable characteristics for practical application. However, most of the previously reported biosensors have used either purified proteins (such as enzymes, metal-binding proteins, or antibodies) or whole cells of genetically engineered microorganisms [15]. These microbial biosensors, which usually use green fluorescent protein or other marker molecules as indicators, face the challenge of toxicity that prevents their use with samples containing high concentrations of heavy metals [16]. Moreover, most of these biosensors cannot provide in situ and real-time Cr(VI) concentration values.

The two compartments of microbial fuel cells (MFCs), namely, the anaerobic and aerobic ones, are a promising approach that can directly utilize microorganisms as biocatalysts for transforming energy as well as to generate power [17]. In recent years, electrical signals have frequently been applied as indicators for the monitoring of heavy metals and nitroaromatic compounds due to their highly speed, sensitivity, and quantifiability [18,19]. Moreover, the potential of MFC-based biosensors for the Cr(VI) detection has been reported. A previous study used a two-chamber MFC as a device for monitoring Cr (VI) in water and highlighted the effect of the co-existing Fe(II) in the system on chromium detection as Fe(II) can reduce Cr(VI) and alter the generated voltage [20], which is unsuitable for in situ detection of chromium. In these MFCs-based biosensors, a correlation among the Cr(VI) concentration and the voltage generation was established with the Cr(VI) acting as electron acceptor at the MFC anode [21]. The results showed its sensitivity to higher Cr(VI) concentrations (2.5–60 mg/L), which preclude its use due to the fact the recommended maximum allowable concentration for Cr(VI) in industrial wastewater based on the Chinese National Standard is 0.5 mg/L. Furthermore, a MFC-based biosensor was developed for Cr(VI) detection using a single strain inoculated in the anode of a MFC [22], but it is not suitable for in situ and real time measurement for the reason that medium refreshment, bacterial cultivation and aseptic techniques must be used, which must be performed in a laboratory.

The Sediment Microbial Fuel Cell (SMFC) concept is a promising alternative technology that comprises an anode and a cathode, in which the anode was embedded in sediment and the cathode is placed above the anode, where it is partly filled with water [23]. Various complex organic compounds such as naphthalene, acenaphthene, phenanthrene [24], and pyrene [25] can be degraded at the anode in SMFCs while sulfate, oxygen and Cr(VI) were reduced at the cathode. Electrons produced by respiration of the microbial community colonizing the anode surface are generated firstly and then transmitted to the cathode through an external circuit [26]. The main advantages of SMFCs over traditional batteries are that they are low cost, of simple construction and they provide long term power output [27]. Thus, this system is applicable as an electron supply device for biosensors for long term and in situ operation. The use of SMFCs as wireless sensors for the real-time detection of environmental and ecological conditions such as temperature and the concentration of dissolved oxygen (DO) was reported [28]. However, there are scarce reports on the development of SMFC as a biosensor for heavy metal monitoring. 

In the present study, we develop an early warning system based on SMFC for long term and in situ monitoring of Cr(VI) concentrations in industrial wastewater treatment processes. A stable performance of SMFC was observed with different temperatures and pH and other co-existing ions. A relationship was obtained depending on the voltage changes in response to different Cr(VI) concentrations. Compared with colorimetric method results, the biosensor for synthesized wastewater and actual wastewater measurement displayed low deviations over 18.3 min. Thus, the system we have developed is suitable and convenient as an early warning device for in situ real time monitoring of the Cr(VI) concentrations in industrial wastewater treatment plants.

## 2. Materials and Methods

### 2.1. Sample Collection

A sewage industrial wastewater treatment plant site situated in Lanzhou, China, (latitude 36°06′ north and longitude 103°39′ east) was selected for sediment sample collection. The samples were collected at a depth of 10 cm beneath the surface and placed in a clean plastic bucket and transported to our laboratory, where they were filtered via a 2-mm strainer for the removal of plant branches as well as leaves and preserved at 4 °C until used. All standards and working solutions such as sodium hydroxide and hydrochloric acid were bought from Sigma-Aldrich (St. Louis, MO, USA) and stored at 4 °C for use in subsequent experiments. 

### 2.2. Biosensor Setup and Operation

**Figure 1:**
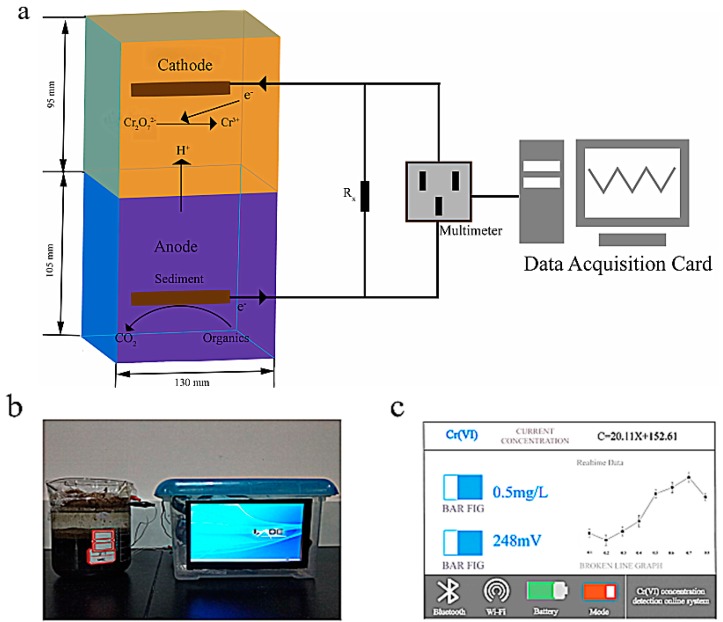
Biosensor design and operation flowchart. (**a**) Schematic diagram of the sediment microbial fuel cell (SMFC)-based biosensor in 3D. (**b**) The operation interface of biosensor. (**c**) The real-time monitoring interface.

Beakers were used to construct the SMFCs. The inner diameter of each SMFC was 130 mm and the height was 200 mm. A sediment chamber and stream sterile water chamber (working volume 1.2 L each) were loaded into the top and bottom of the SMFC, respectively. The sediment was dissolved and cleansed thrice with phosphate-buffered saline. The anode was filled with 1000 mL sediment as fuel and the cathode filled with 1000 mL sterile water containing various concentrations of Cr(VI) and the control used was SMFC reactor without Cr(VI) sterile water in batch mode. In each SMFC, carbon fiber pieces were helpful as positive and negative electrodes (diameter 86 mm × 3 mm). The carbon fiber electrodes were soaked in 1 M HCl and 1 M NaOH to eliminate metals and impurities before use [29,30]. The carbon fiber of the anode was horizontally installed at the center of sediment. The cathode was filled with sterile water and one carbon fiber horizontally placed at 4 cm beyond the sediment-water interface and above the anode. A copper wire functioned in interconnection between the anode and cathode electrodes and an external load (1000 Ω) (Figure 1). Prior to assembly, the SMFCs underwent 15 min of autoclaving for sterilization at 121 °C, and the carbon fiber electrode was also sterilized before use. To ensure the presence of Cr(VI) as electron accepter, the catholyte was filled with nitrogen gas to reduce oxygen before the biosensor work. Dissolved oxygen (DO) was detected using a portable dissolve oxygen meter (JPB-607A, INESA, Shanghai, China). A TOC solid state matrix analyst (varioEL cube; Elementar Analysensysteme, Langenselbold, Germany) was used to quantify the total organic carbon (TOC) content of the sediment. The containers were covered with plastic films to maintain anaerobic conditions and prevent water loss due to evaporation. When the cell voltage had stabilized (at approximately 128 mV) after 18 days of operation, the biofilm in the anode can be seen to take shape. The entire device was placed in a warm room to maintain the temperature of the system. Figure 1a is a 3D figure of the biosensor. Furthermore, we developed a portable device that consisted of a microprogramming control unit (MCU) and a digital signal converter (DSC) for detecting the Cr(VI), Figure 1b shows the operation interface of biosensor. The voltage can be converted to a digital signal by the DSC, and then calculated by the MCU. Figure 1c shows the real-time monitoring interface, which can present the Cr(VI) based on the standard curves with a Wi-Fi or Bluetooth connection.

### 2.3. Analysis and Calculations

During the stabilization period, the SMFC cathode was filled with wastewater. After every 1 min interval the potential (*V*) was automatically recorded using a 8-channel computer-based data acquisition apparatus which was connected to a 1000-Ω external resistor (*R*). The current (*I*) and power (*P*) were calculated as per *I = V/R* and *P = IV* (Ohm’s) equations as described in a previous study [31]. Further, these values were normalized to an apparent surface area of the anode or the working volume. Variations in the external resistor load from 100 to 10,000 Ω, were then used to measure the polarization and power curves, and later when each resistor reached a pseudo-steady state, the voltage was recorded for each [32]. The equation *V = Ecell − IRint*, where *Ecell* is the electric potential of the cell, was used to calculate the internal resistance from the slope of the polarization curve. In situ cyclic voltammetry (CV) tests were helpful in investigating the redox potential of the biosensor, where the SMFC anode was used as working electrode, cathode as counter electrode, and an Ag/AgCl electrode mimicked as reference electrode. The scan rate was 0.025 V/S, and the potential scan rate range used was from −1.5 to 1.4 V. An electrochemical analyzer (CHI604E, Chenhua, Shanghai, China) was used for the analyses. In addition, the diphenyl carbazide method was used to compute the Cr(VI) concentration [33]. The voltage generation of the SMFC can be estimated by the Nernst equation (Equation (1)):

(1) $$E\;=\;E_{0}-\;\frac{RT}{6F}\ln\left(\frac{[Cr_{2}O_{7}{}^{2-}]{\left[H^{+}\right]}^{14}}{{\left[Cr^{3+}\right]}^{2}}\right)$$

*Cr_2_O_7_*^2−^ + 14*H*^+^ + 6*e*^−^ → 2*Cr*^3+^ + 7*H*_2_*O* (*E*_0_ = 1.33 V) (2)

where, *E_0_*—standard electrode potential (V); *R*—gas constant (J/K/mol); *T*—temperature (K); *F*—Faraday constant (C/mol). All experiments were carried out in triplicate.

### 2.4. DNA Sequencing and Analysis

Washing of the anode was done by using phosphate-buffered saline when the operation of the SMFC ended and then microorganisms were collected via centrifugation at 8000 rpm for 10 min. A PowerSoil™ DNA isolation kit which was bought from MO BIO Laboratories (San Diego, CA, USA) was used for extraction of DNA from 0.25 g (wet weight) of anode sediment. DNA concentration was detected by a NanoDrop™ spectrophotometer (Thermo Fisher Scientific, Waltham, MA, USA). The universal primers 515F and 909R were used to boost the V4–V5 hypervariable region of the 16S rRNA gene. The sequencing sample was formulated using a TruSeq DNA kit (Illumina, San Diego, CA, USA). The sequenced data was then organized using the QIIME pipeline version 1.7.0 (http://qiime.org/). All sequence reads were trimmed and assigned to each sample based on their barcodes. For the assemblage of sequences into operational taxonomic units, a 97% identity threshold was used. The Ribosomal Database Project classifier was used to make taxonomic assignations [34]. 

## 3. Results and Discussion

### 3.1. System Condition Optimization 

**Figure 2:**
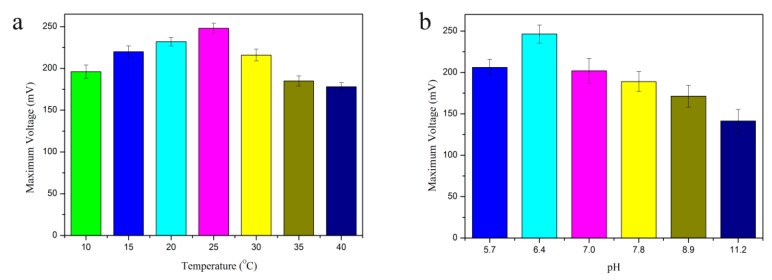
Optimization of the operating parameters. Effects of different temperatures (**a**) (pH 6.4, external resistance 1000 Ω) and pH (**b**) (temperature 25 °C, external resistance 1000 Ω) on voltage generation. Different colored column represent different pH and temperature, respectively. Error bars represent the standard deviation of triplicate tests.

To maximize the signal and assess the execution of the SMFC, the impact of pH and temperature on its performance were determined. We tested the effect of adding 0.5 mg/L Cr(VI) that is Chinese National Standard for industrial wastewater on voltage generation at different pH and temperatures. The maximum voltage changes with varying pH values and temperature conditions are shown in Figure 2.

When the pH was fixed at 6.4, a voltage was generated in the temperature range of 10–40 °C. As seen in Figure 2a, the current increased simultaneously with the temperature in the range of 10 to 25 °C, while a further increase in the temperature to 40 °C reduced the voltage generation. The highest potential of the microorganisms was observed at 25 °C and the maximum voltage generated was 248 ± 0.31 mV. To examine the effect of pH, the temperature was adjusted to 25 °C. The SMFC generated a voltage in the pH range of 5.7–11.2 (Figure 2b). The maximum voltage decreased to 226 ± 0.22 mV at pH 5.7. At pH values below 5.7 or above 11.2, the maximum voltage decreased substantially. The results showed that the highest voltage obtained was 248 ± 0.19 mV at pH 6.4, which was higher than that obtained at other pH conditions.

The pH and temperature are the two factors with the greatest influence on biosensor performance. The reason that the highest potential was measured at 25 °C is probably because that temperature favored the growth of electrochemically active bacteria (EABs) over methanogens and as a result, the loss of charge reduced and the coulomb efficiency increased [35]. It has been reported that an MFC enriched with methanogens exhibited less electricity generation than a MFC enriched with an electrogenic population [36]. The capability of MFCs to transfer organic substrates below 20 °C was reported [37], demonstrating that EABs in such systems can remain active even at lower temperatures. Therefore, this study chose 25 °C to be the optimal temperature for the operation of the SMFC system. The sensor signal and voltage generation were also highly influenced by pH. This could be because of differences in the ion-transferring ability and stability of the biofilm at different pH values. Variations in pH were observed to influence the internal resistance of the MFC. These reports demonstrate that the microbial activity was higher at the optimal pH value than the sub-optimal pH values. The results indicated that an acidic pH was optimal for voltage generation in this system according to Equation (3). This finding is consistent with a previous study using an active pH of 6.5 at the anode and reported maximal current generation by MFC [35]. Therefore, the temperature and pH of 25 °C and 6.4, respectively, were selected as the optimal conditions for sensor operation.

### 3.2. Voltage Generated by the SMFC

**Figure 3:**
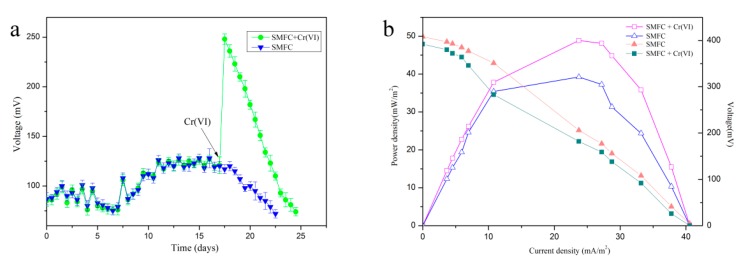
Voltage generation with an external resistance of 1000 Ω in the presence and absence of 0.5 mg/L hexavalent chromium (Cr(VI)) (**a**). (**b**) Polarization and power density curves of SMFC and SMFC with 0.5 mg/L Cr(VI). The current and power density was normalized to the surface area of the carbon fiber.

A combination of sterile water and 0.5 mg/L Cr(VI)-containing water was used in the SMFC. Non Cr(VI)-containing sterile water served as a control. 0–12 days was defined as the startup period. The voltage generation results showed that within 12–18 days, the generated voltage reached a maximum of 128 mV during the control operation, which indicated that the SMFC operated stably during this period. After 18 days of operation, a decrease of voltage was observed. Hence, as per the above observations, the total duration of 18 days was selected for subsequent experiments with water containing 0.5 mg/L Cr(VI), which is Chinese national standard regarded as the safe upper limit for the concentration of Cr(VI) in industrial wastewater. During the experiment with Cr(VI)-containing water added to the cathode in stably period, the voltage of SMFC reached 248 mV quickly and a sharp decrease in voltage was obtained (Figure 3a). The analysis of the power density and polarization curves of SMFC and SMFC with Cr(VI) was done at varying external resistances to evaluate its performance (Figure 3b). 

**Figure 4:**
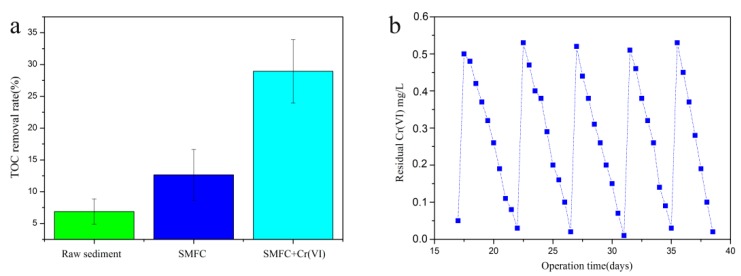
Total organic carbon removal rate of organic substrates in the anode under the optimal operation conditions with different operation (Raw sediment, SMFC and SMFC with 0.5 mg/L Cr(VI)) (**a**). (**b**) Residual concentrations of Cr(VI) in the wastewater of the cathode over five cycles.

The results indicated 408 mV as the peak value for the open-circuit voltage whereas 48.88 mW/m^2^ was the peak value for power density, which was higher than that of the SMFC (39.22 mW/m^2^). Furthermore, based on the slope of the intercept of the linear section of the polarization plot and the equation *R = V/I*, the control group was calculated to 103 Ω, the internal resistance of the system was calculated to be 98.2 Ω, which as the external resistance for subsequent experiments. The SMFC utilizes organic contaminants to generate protons that would be accepted by Cr(VI) in the cathode. The rate of TOC removal (28.93%) was enhanced by 4.21-fold and 2.29-fold as compared with raw sediment (6.87%) and the SMFC without Cr(VI) (12.65%), respectively (Figure 4a). Figure 4b shows that the residual concentration of Cr(VI) in the cathode after 5 cycles of biosensor operation. Cr (VI) reduction rate was approximately 100% in every cycle.

Previous research has reported the development of a two-chambered MFC as a device for monitoring Cr(VI) in water. However, that device could only detect Cr(VI) in acidic water. Earlier in a study, the internal resistance (161 Ω) reported was higher than recorded in the current study (98.2 Ω). For voltage generation, the maximum power density of SMFC with Cr(VI) (48.88 mW/m^2^) was increased compared with the SMFC (39.22 mW/m^2^), which was consistent with a previous study [38]. The occurrence of an increased voltage generation with the addition of Cr(VI) can be described by the Nernst equation (Equation (2)). On the basis of the Nernst equation, the addition of Cr(VI) leads to a higher cathode potential and the voltage and power generation were higher in the SMFC. The baseline voltage generation without the addition of Cr(VI) might be due the presence of oxygen in the cathode. Thus, the increase in voltage generation with the addition of Cr(VI) may be attributable to it having a higher reduction potential than oxygen. Oxygen is a mostly usual electron acceptor in the cathode of a MFC or SMFC. In this study, the voltage generation of the control (SMFC without Cr(VI)) was 128 mV using oxygen as electron acceptor, which is lower than that of the Cr(VI) group, due to the fact the industrial effluent contains some limited amount of dissolved oxygen. Furthermore, oxygen as an electron acceptor at the cathode has been reported but catalysts like Pt, Mn, Fe were needed [39]. Therefore, the oxygen in the industrial effluent has no obvious effect during the system operation. In addition, the increased internal resistance was another reason for the increase in voltage. The internal resistance depends on the electrode material, the presence of a biofilm, and the operation conditions. The shifting of an electron from the cell to the electrode also has a contribution to the internal resistance. Moreover, the shift of electrons from the cell to the electrode occurred easily because of the internal resistance. Therefore, the SMFC reported in this study generated an increased voltage with Cr(VI) as an electron acceptor. The residual concentration of Cr(VI) results showed the Cr (VI) reduction rate was about 100% in every cycle. In this system, the organic substrate is oxidized in the anode and Cr(VI) is reduced at the cathode simultaneously. The electrons were then generated by the organic substrate oxidized in the anode and accepted by the Cr(VI) in cathode. Previous studies have reported that the organic matter of sediment is unlimited when containing sufficient TOC [40]. In addition, the total organic carbon removal rate was 12.65% in a long term operation with 37 days. Thus, the decomposition of organic substrates had no influence on signal detection and the signal decrease must come from the reduction of Cr (VI). The results indicated that the biosensor showed a prominent long-term stability for detecting Cr (VI).

### 3.3. Evaluation of the Biosensor Performance

To get a reliable biosensor, the stability, sensitivity and applicability were evaluated under optimal conditions.

#### 3.3.1. Calibration of the Biosensor

**Figure 5:**
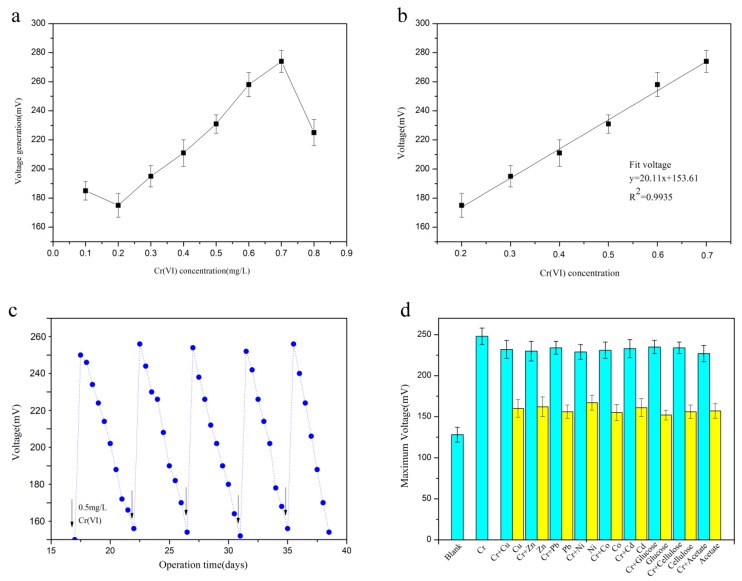
Calibration of the biosensor under the conditions at pH 6.4, temperature of 25 °C and external resistance of 98.2 Ω. (**a**) The maximum voltage at different concentrations of Cr(VI). (**b**) The correlations between the Cr(VI) concentrations and the maximum voltages. (**c**) Voltage generation in the SMFC with the Cr(VI) concentration of 0.5 mg/L over a period of five operational cycles. Arrows indicate the replacement of the catholyte solution with a solution containing 0.5 mg/L Cr(VI). (**d**) Interference experiment design at pH 6.4 and 25 °C with an external resistance of 98.2 Ω. Maximum voltage generation of the SMFC in the presence of various other factors (0.5 mg/L) in the cathode. The standard deviation was representing as error bars.

The system was calibrated using Cr(VI) solutions of various concentrations under the optimal operation conditions. Cathode solutions containing a different concentration of Cr(VI) were used with biosensors in batch mode for establishing the relationship. The results showed that the current was raised in proportion to the molarity of Cr(VI) from 0.2–0.7 mg/L (Figure 5a) and a good linear relationship was observed between the concentration of Cr(VI) and the maximum voltage of the SMFC in the range (R^2^ = 0.9935) (Figure 5b). 

When the Cr(VI) concentration was further concentrated to 0.8 mg/L, the power output decreased and when the concentration of Cr(VI) was set as 0.1 mg/L, this linear relationship was also not consistent. Therefore, 0.2 mg/L concentration was considered the lower detection limit and 0.7 mg/L was observed to be the upper detection limit for the biosensor. The detectable Cr(VI) concentration range is thus around 0.2 to 0.7 mg/L, which the discharge must be below 0.5 mg/L based on the Chinese National Standard, which is included in this range, so the signals of our system can be utilized for monitoring the Cr(VI) concentration in wastewater. The performance characteristics of the SMFC were obtained under the optimal working conditions. The evaluation of the stability of this biosensor was done by using 0.5 mg/L Cr(VI) to test the performance of the SMFC over five cycles. From the results (Figure 5c) it was observed that the system had a stable voltage output with good repeatability. The maximum voltage from the five cycles was 249 ± 2.62 mV, the average response time of each cycle was 18.31 ± 0.25 min, and the average recovery time was 6.42 ± 0.36 h in each cycle. To investigate the accuracy of the system, sterile wastewater samples were obtained from a chemical industrial plant without Cr(VI) and washed thrice with phosphate buffer. Different heavy metals, including Cu^2+^, Zn^2+^, and Pb^2+^, etc., which were at the same concentration (0.5 mg/L) as Cr(VI) and some organic compounds were added to the cathode along with 0.5 mg/L Cr(VI) to evaluate the accuracy of the SMFC system and the maximum voltage generation in the presence of the various additives was 266 ± 0.26 mV, which showed not influenced by other factors Furthermore, the addition of different heavy metals at the same concentration as Cr(VI) into the cathode alone without Cr(VI) were also investigated, and the results also demonstrated that there was no effect on the voltage generation of system (Figure 5d), which is similar to the result obtained when 0.5 mg/L Cr(VI) alone was present. A stable performance of the system was obtained, which proved that the SMFC-based biosensor has high potential for Cr(VI) measurement in industrial wastewaters.

#### 3.3.2. Application of the Biosensor in Actual Wastewater Treatment

It is very important to test the biosensor in the actual detection of industrial wastewater. A system calibration step was performed to evaluate the stabilities of the sensor response and the lower detection limit. Different concentrations of synthesized wastewaters (prepared by dissolving appropriate amounts of NaCl, NH_4_NO_3_ and NH_4_Cl in sterilized tap water) were prepared and some out on range samples were appropriately diluted before detection using our SMFC-based biosensor. Actual wastewater samples were collected from the Lanzhou Petrochemical Company. The operational conditions that showed the best performance in terms of stability and accuracy were used. To evaluate the practicability of the biosensor for measuring Cr(VI) concentration in industrial wastewater, solutions containing Cr(VI) at several concentrations ranging between 0.2–0.7 mg/L were measured using both the biosensor and a spectrophotometer (Table 1). The D-value using the biosensor for detecting the Cr(VI) concentration in synthesized wastewater compared with the standard Cr(VI) concentration was lower (<0.03). Compared with the relationships between the Cr(VI) concentration and maximum voltage that were determined using those two methods showed no significant differences, the deviation of the two methods was less than 5%, when the detected concentration was in the range from 0.2–0.7 mg/L. Three actual wastewater samples containing different Cr(VI) concentration (denoted as a, b, c) were also detected using the biosensor method and colorimetric method. As listed in Table 1, the biosensor detection results presented a lower deviation (<8%) compared with the colorimetric method. These results indicate that the biosensor had an stable real time capacity for detecting Cr(VI) between the range from 0.2–0.7 mg/L in a short time of 18.3 min.

To date, a number of biosensors have been developed for Cr(VI) detection such as enzyme biosensors [41], and amperometric microbial biosensors [14]. Nevertheless, the current biosensors for heavy metal monitoring require expensive reagents like proton exchange membranes (PEMs), and have low selectivity, implying high-cost and low-output., which makes them not suitable for Cr(VI) detection. Our designed system exhibited excellent characteristics for monitoring actual Cr(VI)-contaminated wastewaters without complicated processes or expensive equipment. High accuracy is an important performance feature for a biosensor to applied to actual Cr(VI)-containing wastewater measurement. As the tabulated results showed, using the colorimetric method is slightly more accurate than the SMFC-based biosensor, but it also has some differences in accuracy. Low deviation (8%) was obtained with our system, which was 2% lower than in a previous study [21]. Therefore, this SMFC-based biosensor can be developed as an early warning system for monitoring Cr(VI) concentrations in industrial wastewater treatment plants.

### 3.4. Scanning Electron Microscopy and Cyclic Voltammetry Analysis

**Figure 6:**
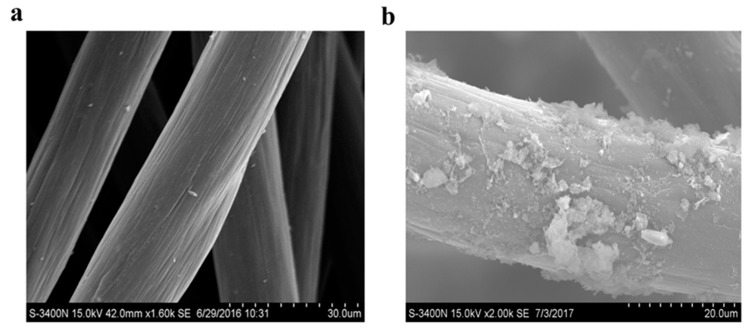

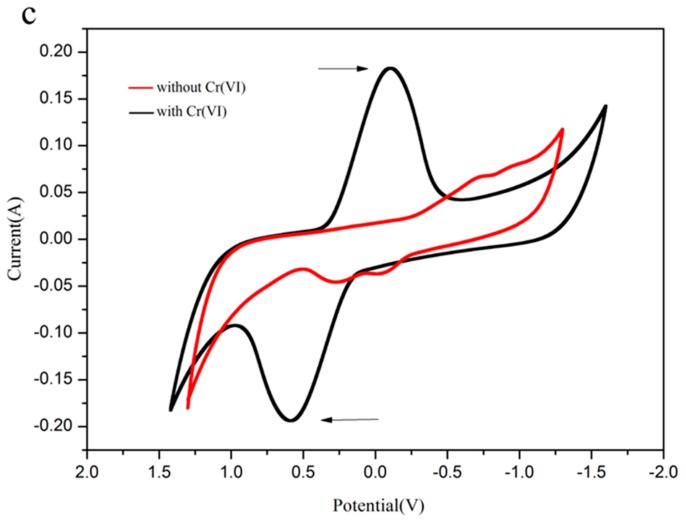
Scanning electron microscope images of carbon felt electrodes showing the surface of the anode electrodes before (**a**) and after (**b**) treating actual wastewater. Cyclic voltammetry (CV) curves for SMFC of control and SMFC with Cr(VI) (**c**). Scanning rate was 0.025 V s^−1^.

Scanning electron microscopy and cyclic voltammetry analysis were used to assess the electrochemical activity. The bacterial cells are attached and fixed on the surface of the anode, as a biocatalyst, which can degrade the substrate and generate the current [42]. The surface of the anode (carbon fiber) before and after treating actual wastewater was visualized by a Scanning Electron Microscope (SEM, JXA-840A, JEOL, Tokyo, Japan) to determine the microbial attachment and formation of a biofilm on the anode electrode surface. The SEM results showed the surface of the electrodes was covered with the biofilms containing electrochemically active bacteria (EAB) (Figure 6a). It was observed that the anode had a rough and porous surface due to the attachment of the microbes, thereby helping to improve the direct electron transfer in the SMFC, whereas the control anode without microbes had a smooth and clean surface (Figure 6b). This would explain the differences in electricity output.

The extracellular electron transfer (EET) mechanism is strongly dependent on the redox activity in the SMFC system and is required to generate electrical conductivity in the biofilm and electrode due to the fact the biofilm conductivity itself is not great enough to transfer electrons fluently to/from the electrodes [43]. To reveal the underlying mechanism of EET, cyclic voltammetry (CV) was used at a scan rate of 0.025 V/s. There were no apparent oxidation-reduction peaks in the control group (without Cr(VI)). According to the CV curve results, a pair of oxidation/reduction peaks were achieved with the cathode solution with Cr(VI) at −111 mV and 581 mV, respectively (Figure 6c), proving the importance of biofilms responsible for the electrochemically catalytic activities. The diffusion process is a rate-limiting step of the shuttle-mediated EET. The Cr(VI) concentration could finally enhance the electron shuttle-mediated EET. These results demonstrated that cathode solution with Cr(VI) showed higher redox potential than without Cr(VI).

### 3.5. Microbial Community Analysis

**Figure 7:**
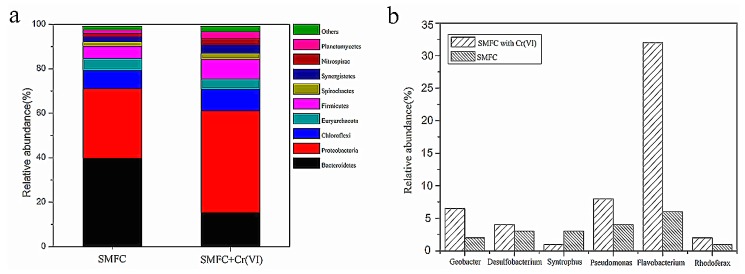
Relative abundance of microbial communities structure of biofilms from the Sediment MFC and Sediment MFC with Cr(VI) at the phylum level (**a**) and genus level (**b**).

MiSeq sequencing of the 16S rRNA gene was applied to analyze the diversity of the microbial communities in the biofilm of the anode. To establish a relationship between electricity production and the microbial community, the anodic biofilm microbial communities of the SMFC were examined with or without the addition of Cr(VI) to the cathode after treating actual wastewaters. The results represented that the microbial communities can degrade organic substrate at the anode and Cr(VI) is reduced at the cathode. As seen in Figure 7a, *Proteobacteria* was the main population in the SMFC (31.8%) or SMFC + Cr (VI) (45.8%), which showed 44.0% higher in SMFC + Cr (VI) than that in SMFC, and *Bacteroidetes* was the second most abundant at the phylum level. This is consistent with previous studies which also found *Proteobacteria* were the predominant bacteria in the anode of MFCs [44]. Next, we analyzed the data at the genus level. From Figure 7b, we found that *Flavobacterium* (32%) was specifically increased and gradually became the predominant bacteria due to the fact the other species could not tolerate the toxicity of Cr(VI), which is consistent with a previous study [45]. In addition, several common electrogenic bacterial genera, including *Geobacter*, *Pseudomonas*, and *Rhodoferax* were enriched, indicating that these microorganisms contributed to electricity generation with the addition of Cr(VI). It is well-known that most exoelectrogens such as *Geobacter* and *Pseudomonas* belong to the *Proteobacteria,* so the result was consistent with the present study. 

EABs have a major importance in MFCs as they catalyzes the oxidation of organic substrates to generate power [46]. According to a previous study, EABs are responsible for voltage generation and electron transfer in SMFCs [47]. The microbiota in the SMFC differed from that in SMFC and was particularly enriched in *Flavobacterium*, which are specialists that tolerate the toxicity of Cr(VI). *Proteobacteria* was found as the dominant EAB phylum and its abundance was 45.8% higher in the SMFC with Cr(VI) than in the SMFC, representing a 1.44-fold increase. The operation of the SMFC with Cr(VI) resulted in an increased abundance of *Proteobacteria*, which corresponds to the results of previous studies [48,49]. Members of the phylum *Proteobacteria* have often been found to be enriched on MFC anodes, where they contribute to the current generation [49]. These results show that the phylogenetic diversity in the microbial communities of anodic biofilms with added Cr(VI) differed from those in SMFC, in agreement with the findings of previous studies. The non-EAB organisms are hypothesized to degrade polymers and generate electron donors for EABs.

## 4. Conclusions

In the present study we describe an SMFC that functions as a biosensor for real time in situ detection of Cr(VI) concentrations. Excellent selectivity, accuracy and low deviation under various conditions of pH and temperature were obtained, and co-existing ions also did not affect the stability of biosensor, which indicated that the biosensor showed high reliability as an early warning system for Cr(VI) determination in industrial wastewaters. A good relationship between the maximum voltage value of biosensor and the different Cr(VI) concentration (0.2–0.7 mg/L) was obtained, whereas the Chinese National Standard about Cr(VI) concentration in industrial wastewater is 0.5 mg/L. Therefore, this biosensor could be useful as an early warning system for monitoring Cr(VI) in industrial wastewater treatment plants. 

## Figures and Tables

**Table 1:** Application of biosensor and colorimetric method for Cr(VI) measurement in synthesized wastewater and real wastewater.

	Synthesized Wastewater						Real Wastewater		
	Standard Cr(VI) Concentration (mg/L)								
	0.25	0.35	0.45	0.55	0.65	0.70	a	b	c
Colorimetric method	0.24 ± 0.78	0.35 ± 1.02	0.46 ± 2.04	0.55 ± 0.68	0.64 ± 1.33	0.71 ± 2.34	0.46 ± 3.54	5.26 ± 4.32	24.63 ± 1.62
SMFC-Biosensor	0.25 ± 1.24	0.34 ± 2.23	0.47 ± 1.98	0.54 ± 0.86	0.67 ± 2.01	0.69 ± 1.08	0.48 ± 2.42	4.89 ± 2.07	26.18 ± 3.42
D-value (C)	0.01	-	0.01	-	0.01	0.01	-	-	-
D-value (S)	0.01	0.01	0.02	0.01	0.02	0.01	-	-	-
Deviation (%)	4.17	2.86	2.17	1.82	4.69	2.82	4.35	7.03	6.29

D-value (C): Deviation value of Colorimetric method; D-value (S): Deviation value of SMFC-based biosensor; Both of D-value (C) and D-value (S) were absolute value; Deviation (%): The detect value using biosensor compared to colorimetric method. a, b, c: name of three actual wastewater samples.
